# Healing Environment in Pediatric Cancer Centers by Utilizing Positive Distractions

**DOI:** 10.3390/bs14111010

**Published:** 2024-10-30

**Authors:** Aseel Alhsainat, Kağan Günçe

**Affiliations:** Faculty of Architecture, Eastern Mediterranean University, North Cyprus Via Mersin 10, 99628 Famagusta, Turkey; kagan.gunce@emu.edu.tr

**Keywords:** healing environment, perception, positive distraction, children, cancer care facilities

## Abstract

In recent years, a broader perspective has viewed the use of therapeutic environments in healthcare, transforming the hospital’s environment into an energizing atmosphere that benefits both employees and patients. Scientists found that exposure to natural elements like nature scenes, sunlight, art, calming colors, and soothing sounds significantly sped up healing and created a more relaxed hospital environment. This research emphasized the importance of healthcare settings’ interior design in promoting patient well-being through beneficial distractions. The motivation for this investigation came from the need to develop an understanding of positive distractions in the cancer care environment and showed how this understanding could be used to enhance the space experience to promote health and well-being. This research adopted a mixed-methods approach. First, a qualitative method using a critical review of the literature was used to effectively present, analyze, and synthesize literature from diverse sources, followed by a quasi-experimental research method. This research is intended to focus on the attributes of positive distraction as one of the psychosocially and psychologically supportive healthcare design aspects that evoke positive feelings in children’s cancer center experiences.

## 1. Introduction

Ghazali and Abbas [1] trace the origins of healing environments back approximately 2300 years. Huelat [2] expands this concept to include holistic and spiritual elements within complementary and alternative medicine. Recent research in healthcare design underscores the profound impact of physical environmental factors on well-being. As demanded, healthcare environments are designed to promote patient well-being, which results in enhanced health outcomes [3].

### 1.1. Overview

Recent research highlights the importance of designing hospital spaces that minimize patient stress and anxiety while promoting recovery. For example, Ulrich et al. (2008) [3] present empirical evidence demonstrating how exposure to nature, art, and calming colors significantly improves patient outcomes. These environmental elements, often termed as positive distractions, divert patients’ attention from stressors, thus reducing anxiety and enhancing emotional well-being.

#### 1.1.1. Theories Underpinning Healing Environments

**Figure 1:**
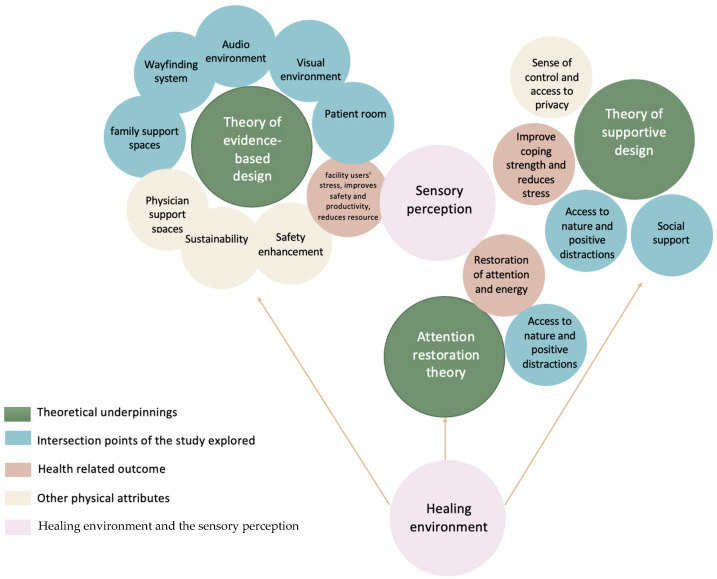
Theoretical framework of the current study.

The theoretical frameworks that underpin the concept of healing environments include evidence-based design (EBD) [4], supportive design theory [5], and attention restoration theory [6], as shown in Figure 1. Each of these theories offers distinct insights into how built environments can influence patient health.

The consolidation of these pre-existing theories and keywords derived allowed for a methodology to be produced to explore physical healthcare environments considering positive distractions, placing the user experience at the forefront. This investigation concentrated on the intersecting attributes of these theories that have promoted supportive design for a positive patient experience, correlated with health concerns. Defending the notable design factors and keywords associated with these theories in relation to the selected patient group in this research aided in the development of the conceptual framework that this study proposed.

##### Evidence Based Design

Evidence-based design (EBD) has been extensively recorded as the healing environment in healthcare. EBD is an approach grounded in the application of scientific research to guide architectural and interior design decisions in healthcare settings. This theory serves as a foundation for understanding the importance of integrating environmental features that promote healing. The discipline of evidence-based design (EBD) has progressed over the past twenty-five years. It applies scientific knowledge and methods to help guide healthcare facility design in a manner that reduces facility users’ stress, improves safety and productivity, reduces resource waste, and enhances sustainability [7]. There is a growing body of evidence that supports the idea that the physical environment of healthcare settings, including their layout, materials, equipment, and furnishings, plays a significant role in either facilitating or preventing the spread of pathogens [8]. The movement towards EBD in health care started with the concept that patients who viewed trees had shorter postoperative stays, took fewer pain relief drugs, and held a favorable response about their outcomes in medical notes when compared to those exposed to a view of a brick wall [9].

In line with this, Mackrill [10] observed that enhancing healthcare services and environments through positive design has a significant influence on the physical and mental well-being of both patients and staff. These improvements have involved a range of non-pharmacological approaches to improve the discomfort associated with stress and the duration of stay in healthcare environments [11]. For instance, the implementation of therapeutic gardens and healing gardens (HG) as non-pharmacological measures has demonstrated their efficacy in enhancing overall well-being and alleviating pain, restlessness, and distress among patients in healthcare environments [12].

##### Supportive Design Theory

The supportive design theory, proposed by Ulrich (1991) [5], suggests that healthcare environments should reduce environmental stressors and instead offer elements that foster a sense of control, provide social support, and offer positive distractions. This theory is critical in understanding the role of the environment in improving psychological outcomes for patients, particularly in pediatric care, where emotional well-being is closely tied to recovery.

The concept of supportive design explores the impact of the healthcare physical-social environment on the health and well-being of patients, with a focus on reducing stress-related issues to improve the overall hospital experience [5]. This theory is well documented and is often used to describe and interpret patients’ needs or to suggest strategies or approaches for achieving supportive design within the hospital premises. The supportive design theory offers a potential framework for enhancing overall health promotion through design in both the physical environment and healthcare facilities. This theory highlights the significance of the physical environment in efficiently fostering improved health outcomes by eliminating stress-inducing factors, which frequently have adverse effects on health outcomes [5,13].

According to Ulrich, healthcare environments that promote well-being are those that provide a sense of control over surroundings, access to social support, and positive distractions. This theory, widely accepted in the field, is frequently utilized to understand patients’ requirements and recommend strategies for supportive design [14].

These are the three main overarching precepts of the theory, which explain the attributes that provide for the patient’s psychological needs, family members, and caregivers, such as reduced stress and increased coping abilities in healthcare facilities [5,13,15].

1. Social Support

In an environment that may be unfamiliar and filled with stress, the presence of social support from others can help to alleviate stress levels [16]. Extensive research has consistently shown that individuals who receive ample social support tend to experience lower levels of stress and enjoy better overall health compared to those who are socially isolated [15]. Consequently, social support can be defined as the provision of emotional assistance and care to an individual, as well as the support received from others.

2. Sense of Control

According to Carver [17], the concept of “control” refers to an individual’s actual or perceived ability to understand and manage their actions, regulate their circumstances, and determine the influence of others’ behaviors and perceptions. Several studies have indicated that individuals who believe they have some level of control over their situation exhibit stronger stress-coping abilities compared to those who perceive a lack of control [13]. The perception of control over one’s surroundings serves as a potential mechanism in the association between environmental choices and well-being, as well as stress reduction [18]. Specifically, the sense of control is linked to opportunities for modifying or adjusting various aspects of the environment [19]. To address this issue, it is imperative to implement a critical supportive design strategy that fosters a more controlled environment.

Research has shown that architectural designs play a significant role in the loss of control experienced by individuals in healthcare settings. For instance, rooms that obstruct patients’ view of the outside, compel bedridden patients to gaze at harsh ceiling lights, or lack clear signage for wayfinding contribute to this problem [20]. In certain medical conditions, such as cancer, individuals may experience a loss of control over their own bodies. In such cases, providing opportunities for patients to have some control over their physical surroundings can be beneficial in combating the feelings of helplessness they may be experiencing.

3. Positive Distractions

The utilization of positive distractions is a key aspect of the patient-centered care model, as highlighted by Frampton and Clancy [21]. Within the healthcare setting, the concept of positive distraction has been extensively studied and acknowledged for its beneficial effects [3,4,5,7,9,22,23]. These distractions serve to redirect individuals’ attention away from their discomfort and anxiety, allowing them to focus on alternative stimuli.

Positive distractions are elements of environmental and social factors that are acknowledged for their ability to improve overall health and reduce stress levels effectively. These distractions include art, being in nature, music, and other calming sounds, engaging in recreational activities, and participating in social interactions. Ulrich [22] highlights the importance of these positive distractions in enhancing well-being. Furthermore, Ulrich’s research [13] shows that individuals dealing with anxiety or stress-related issues experience positive outcomes when exposed to certain natural environments, leading to faster recovery rates.

##### Attention Restoration Theory

Kaplan and Kaplan’s (1989) [24] attention restoration theory (ART) posits that natural environments, or representations of nature, have the capacity to restore cognitive functioning and reduce mental fatigue. This theory has been widely applied in healthcare settings to justify the inclusion of nature-based elements, such as gardens and natural scenery, which contribute to the reduction of stress and the enhancement of mental clarity in patients.

The attention restoration theory (ART) posits that individuals can derive advantages from opportunities to (1) temporarily distance themselves from the pressures of daily life, (2) immerse themselves in vast environments and situations (“extent”), (3) participate in activities that align with their inherent motivations, and (4) actively engage with stimuli that possess a gentle allure [6]. This confluence of factors encourages “involuntary” or “indirect” attention while allowing our “voluntary” or “directed” attention capacities to recover and restore [25]. Relaxing settings (such as places of worship) and activities (such as sleep) may provide restorative opportunities, but ART argues that nature may be beneficial because it has an “aesthetic advantage” [24,26].

This research described the selected theories used to underpin the study. The theories included evidence-based design, supportive design, and the attention restoration theory. The justification for choosing these theories was grounded in the premise that they are all geared towards transforming the built environment through environmental design factors. They all attempt to improve wellness for users in a healthcare environment and incorporate environmental, social, technological, and psychological aspects to enhance the quality of healthcare facility design. Lastly, they all employ connections between healthcare and positive distractions to provide a positive environmental design for patients. Understanding the relationship between these theories and how they contribute to the improvement of wellness and the architectural design quality of care facilities provides a theoretical standpoint to critically investigate how well these theories have aided research on the topic under investigation.

#### 1.1.2. Impact of Positive Distractions on Patient Outcomes

In healthcare environmental research, since the 1970s, there has been a notable shift in emphasis from mitigating the adverse effects of the physical environment to establishing environments conducive to fostering positive experiences [27]. In accordance with the principles of patient-centered care, the design concept of positive distraction was developed in response to a surge in research into the effects of stress in healthcare environments [5,13]. A positive distraction is defined as an environmental element or circumstance that elicits favorable emotions, sustains interest and focus, and ultimately promotes advantageous physiological and/or psychological transformations [5].

The term positive distractions refers to environmental features that help divert patients’ attention away from stressors associated with illness or hospitalization. Studies, such as those conducted by Ulrich, demonstrate that elements like nature scenes, soothing music, and interactive art installations can significantly improve patient outcomes by lowering blood pressure, reducing the perception of pain, and improving overall mood. The implementation of positive distractions within healthcare settings has been shown to alleviate patients’ stress and improve their mood, consequently promoting the process of recovery, although it can yield positive effects on the physical and mental health of individuals [5,22,28]. As a result, design processes can be modified to more effectively integrate positive distraction features that permit and calm occupants, as opposed to the outcomes produced by bare walls and small windows.

**Figure 2:**
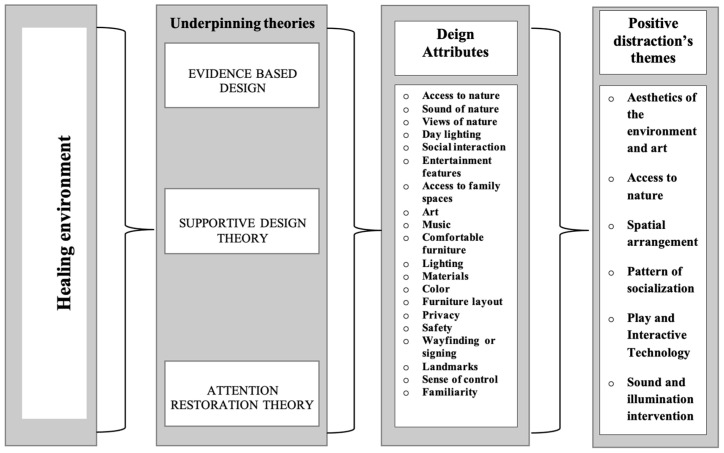
Positive distraction themes highlighted from underpinning theories in the research.

Distraction is a common coping mechanism employed by children to deflect their attention from distressing stimuli. Distraction can be described as “sensory shielding”, in which an increased influx of sensory information from alternative sources serves to obscure the patient’s perception of pain [29]. Positive distractions are specific categories of environmental elements that have been empirically demonstrated to effectively alleviate tension and foster well-being. Six positive distraction themes were discerned in this study: aesthetics of the environment and art; access to nature; spatial arrangement; patterns of socialization; play and interactive technologies; and sound and illumination interventions. The order of the six themes was as follows: broad design elements of the constructed environment came first, followed by interior features, and finally design details and sensory stimulations. Changes in many health indicators and experiences, including stress, anxiety, physiological arousal, pain and distress, levels of comfort and calm behaviors, emotional status, recovery, control or familiarity, social support, sleep, waiting experience, levels of physical activity and contact with nature, patient satisfaction, were discovered to be associated with these themes. The subsequent summary provides a synopsis of the examined publications. Figure 2 illustrates the selected theories from the current study, the design attributes associated with these theories, and the themes of positive distractions derived from these design attributes.

##### Aesthetics of the Environment and Art

**Figure 3:**
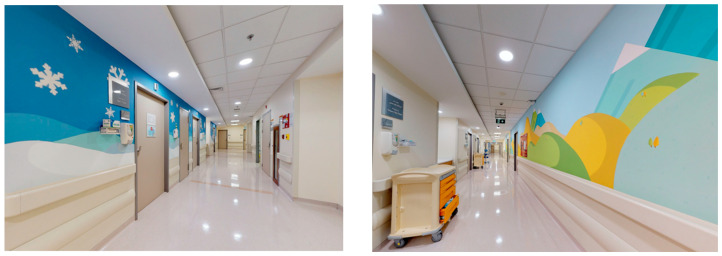
Art in King Hussein Cancer Center corridors in Amman, Jordan.

Research has shown that adult patients respond more positively to realistic artwork representing nature compared to abstract art [13,30]. This finding appears to apply in regard to pediatric patients as well, irrespective of their cognitive development stage [22]. Particularly, there was a widespread preference for hospital designs for children that incorporated natural elements, such as views of the ocean and shore [31]. Overall, pediatric patients exhibited a preference for environments that incorporated a diverse range of colors, thematic artwork that was suitable for their age, and an aesthetic that avoided resembling that of a hospital [32]. There exists a widespread preference for hospital designs that favor the creation of a visually appealing setting, which has the potential to alleviate patients’ anxiety and tension while also enhancing their healthcare experience and overall satisfaction. Figure 3 shows artworks displayed in the corridors of King Hussein Cancer Center, Amman, Jordan.

##### Access to Nature

**Figure 4:**
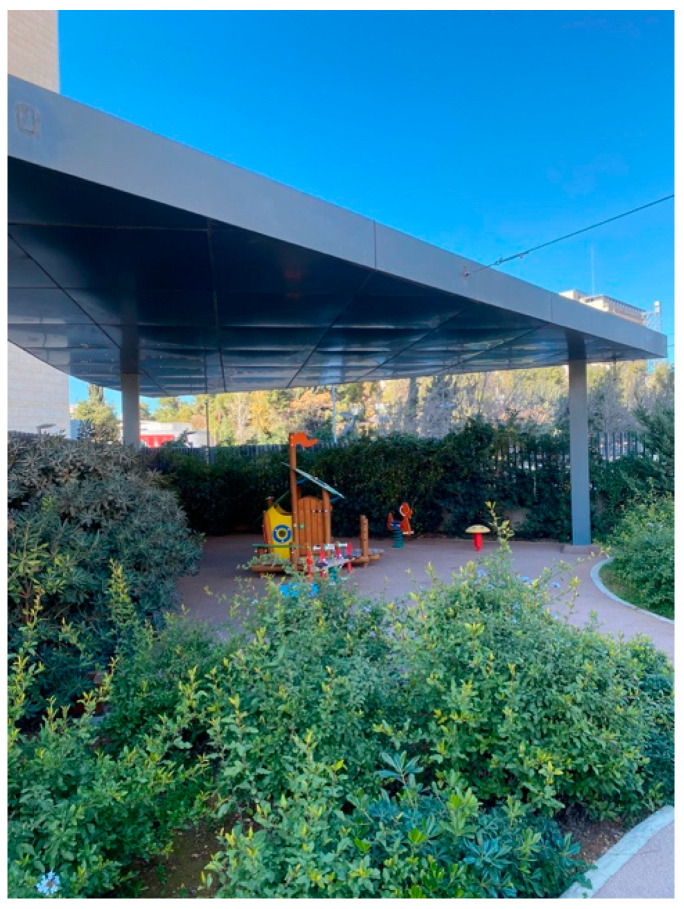
Nature in King Hussein Cancer Center in Amman, Jordan.

Whitehouse and his colleagues [33] introduced researchers to the concept of healing gardens in pediatric healthcare settings. They discovered that the garden space was seen as a place of restoration and healing, and that garden use was associated with increased consumer satisfaction, improved emotional respite for visitors, and decreased pain and suffering [23,33]. A variety of seating based on the scale of the occupying children, enhanced accessibility and circulation, additional trees, a pleasant microclimate, playful elements, and security and privacy are all design elements that may encourage children to utilize such a garden [23,34]. Several researchers whose studies have focused on the function of nature in healthcare settings have discovered that being in close contact with nature, even through a window overlooking a garden, enhances cognitive functions, restores concentration, and promotes psychological well-being [4,6,24]. Figure 4 shows the garden of King Hussein Cancer Center.

##### Spatial Arrangement

An interview was conducted by Adams with child participants to ascertain their perspectives on the central atrium areas of a pediatric hospital. It was determined that the architectural scale of the spatial design influenced the perceived intimacy of the space among pediatric patients [35]. The public atrium provides distractions while improving chances for social interaction and is linked to the emotional well-being of young patients [36]. It has been determined that enhancing character, innovation, and access in the design of pediatric healthcare facilities can improve the patient experience through healthcare buildings and spatial layout.

##### Pattern of Socialization

**Figure 5:**
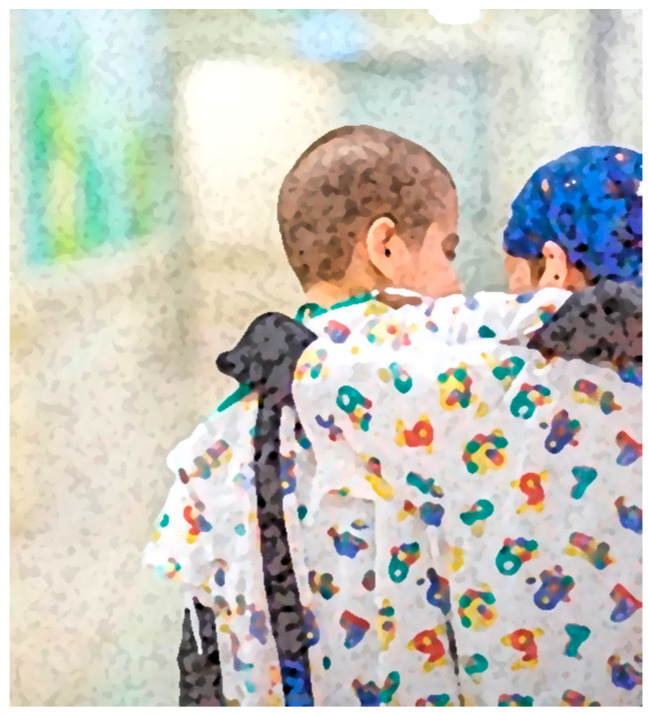
Two patients walking in the corridor of King Hussein Cancer Center in Amman, Jordan, demonstrating a pattern of socialization.

Lambert et al. discovered that children ages 5 to 8 who were hospitalized exhibited a profound sense of isolation [37]. They desired to experience a sense of social connection with both the hospital’s staff and the outside world. Social support has been found to decrease stress in many groups of people [5]. Each age group should have the opportunity to socialize in a designated space. Zimring et al. highlighted patient support, privacy and control, and the availability of social contact with family, friends, and even other patients as essential components of the social environment. Maintaining social connections provides substantial therapeutic benefits. Patients experience significant advantages from receiving social and parental support during their rehabilitation from illness. Research suggests that furnishing lounges and waiting areas with comfortable, portable chairs placed in small, adaptable groups can enhance social interaction [38]. Figure 5 depicts two pediatric patients walking and engaging in a conversation in the corridor of King Hussein Cancer Center.

##### Play and Interactive Technology

**Figure 6:**
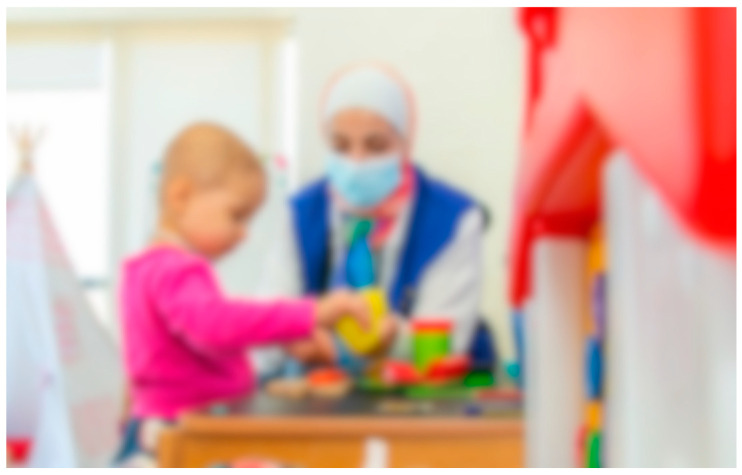
A child plays in the play area while she is staying at King Hussein Cancer Center in Amman, Jordan.

Pediatric patients who engage in play and interactive technologies have been shown to experience less pain and suffering during medical procedures, as well as less anxiety, arousal, and a calm demeanor. According to a study, displaying multi-sensory stimuli alongside ambient artwork may aid in calming down pediatric patients and maintaining their focus during wait times [39]. During hard medical treatments, virtual reality could be a useful diversion [40]. Furthermore, it has been demonstrated that nature-related content, such as animals, aquariums, and zoos, can successfully divert young patients’ attention and reduce their feelings of anxiety [39,40,41]. Health professionals in pediatric healthcare facilities view “play” as an activity that assists children in utilizing waiting times more effectively, reducing anxiety, and improving interactions and communication with health professionals, as shown in Figure 6 [42].

##### Sound and Illumination Interventions

It has been realized that natural sound interventions have therapeutic effects, as they reduce noise, promote tranquility, and calm the behaviors of adolescent patients in waiting areas [43]. Watts and colleagues discovered that music can contribute to well-being compared to natural sounds. They highlighted that choice significantly impacts how effective music is as a beneficial distraction. Conversely, natural sounds, particularly water sounds, were commonly seen as a beneficial form of distraction. Technology has expanded the possibilities for sound and lighting interventions in healthcare design, such as using colored ambient lighting to match the subject of animation and art. Quan and colleagues discovered that ambient lighting and animation interventions in a radiographic scan setting could help shorten medical procedures, increase parental satisfaction, and possibly reduce patient stress [44].

As a summary, Table 1 shows the connection between the design factors of positive distractions in healthcare and the resulting health outcome.

### 1.2. Research Question

This research seeks to answer this question:

In children cancer care environments, how can positive distraction be better incorporated to evoke a positive influence on the patient’s experience?

This question was inspired by a desire to understand how positive distractions can influence children’s space experiences in cancer center care facilities.

### 1.3. Research Aim and Objective

This study examines how children interact with and respond to their surroundings, emphasizing the importance of understanding their physical, social, and cognitive needs. It explores the role of the physical environment in hospital settings, with a particular focus on enhancing the health and well-being of children. This research seeks to deepen our understanding of the role of positive distractions in cancer care settings, particularly focusing on improving the overall experience and well-being of pediatric patients. The study effectively establishes a theoretical framework highlighting the importance of positive distractions in enhancing the care environment for young patients. By integrating principles from supportive design, evidence-based design for healthcare, and attention restoration theory, this research provides a robust foundation for identifying and implementing design features that contribute to the well-being of children undergoing cancer treatment.

## 2. Research Methodology

This study employed a quasi-experimental design to evaluate the impact of positive distraction features on the patient experience in pediatric cancer care. The study’s design addressed both the practical and ethical challenges of researching pediatric patients, focusing on how positive distractions in the healthcare environment can enhance well-being.

### 2.1. Participant Selection

The study included a sample of 20 pediatric patients receiving treatment at the King Hussein Cancer Center (KHCC) in Amman, Jordan. Given the sample size, this study has been categorized as a pilot study. This helps to assess the feasibility of the interventions before larger-scale implementation.

### 2.2. Data Collection Methods

#### 2.2.1. Data Collection Will Occur in Two Phases

##### Quasi-Experimental Design

The quasi-experimental design involved implementing specific design features at KHCC, Amman, Jordan. Patient outcomes were measured using standardized assessment tools to evaluate well-being, satisfaction, and emotional responses to the environment. Due to the nature of the study, randomization was not feasible; therefore, a quasi-experimental study design was utilized to assess the impact of positive distractions on the emotional and psychological well-being of pediatric patients. This approach allowed for a comparative analysis between two distinct groups: an intervention group exposed to a high-intervention setting that incorporated various positive distraction elements, and a control group situated in a low-intervention environment. The quasi-experimental design enabled the collection of both qualitative and quantitative data, facilitating a comprehensive evaluation of patient outcomes. Surveys and assessments were administered to measure satisfaction levels, emotional responses, and overall well-being across both groups. This methodological approach provided valuable insights into how environmental factors influenced patient experiences and the effectiveness of therapeutic design elements.

##### Statistical Analysis

A comprehensive statistical analysis was performed to evaluate the relationship between the implemented design features and their effectiveness. Specifically, a t-test was utilized to analyze differences in outcomes, ensuring the accuracy of the findings while controlling for potential confounding variables.

### 2.3. Literature Review

A critical review of the literature preceded the quasi-experimental phase to identify relevant studies on positive distractions in healthcare design. This review involved a systematic search using mainstream medical and architectural databases, focusing on these keywords: (1) pediatric, children, youth, young patient, adolescent, and teenager; (2) hospital, healthcare environment/setting/facility, clinic, and pediatric center; (3) public space/area, waiting room/area, hallway, playground, playroom, and garden; (4) stress, emotion, anxiety, mood, behavior, perception, pain, distress, experience, and satisfaction; and (5) art, color, play, image, video, game, distraction/distractor, and technology. The major search terms and phrases identified in this study included, but were not limited to, “evidence-based design”, “supportive design theory’ “healthcare”, “attention restoration theory”, “hospital environment”, “children’s”, “cancer” and other relevant key words derived from materials retrieved. A wide variety of databases were utilized, including the ProQuest Database, Springer Publishers, PubMed’s search engine, MEDLINE, the PsycINFO database by the American Psychological Association, and Google Scholar. The Center for Health Design research repositories and the International Academy for Design and Health resource library were also searched.

A thematic analysis will summarize findings from the literature, providing insights into existing knowledge and informing the design features tested in the quasi-experimental phase.

### 2.4. Integration of Qualitative Insights

While this study primarily focused on the quasi-experimental design, the qualitative insights gained from the literature review guided the selection of specific environmental elements to implement. This integration ensured that the study was rooted in existing evidence while also addressing the unique needs of pediatric patients.

### 2.5. Compliance with Guidelines

The study employed a quasi-experimental design primarily as a feasibility study to assess the practicality of implementing positive distractions in a clinical setting. This approach allowed for comparative analysis between two distinct groups: an intervention group exposed to a high-intervention setting with various positive distraction elements, and a control group in a low-intervention environment.

In line with the principles of a feasibility study, the research evaluated both the feasibility of the intervention and its potential effectiveness. Surveys and assessments measured satisfaction levels, emotional responses, and overall well-being across both groups. A checklist guided the study’s design and implementation, addressing essential components such as participant selection, randomization, blinding, and outcome measures. Understanding the relationship between these theories and their contributions to improving wellness and the architectural design quality of care facilities provided a theoretical basis for investigating their impact on the topic.

Qualitative methods included interviews with parents and healthcare professionals, offering rich insights into the experiences of pediatric patients in different hospital environments. As this was a pilot study, it adhered to relevant guidelines for feasibility studies, including the CONSORT statement for pilot trials. A retrospective trial registration was completed to ensure transparency and rigor in the research process.

## 3. Exploring the Role of Positive Distractions in King Hussein Cancer Center

### 3.1. Hypothesis

Positive distractions within cancer center environments significantly enhance the well-being of pediatric patients, thereby facilitating improved healing conditions.

### 3.2. Aims and Objective

The goal of this study was to investigate the effects of positive distractions on the health of pediatric patients at King Hussein Cancer Center (KHCC). The study focused on the beneficial impacts of hospital interiors on patient rehabilitation, assessing current conditions and design trends for creating a therapeutic environment that ensures patient satisfaction. It emphasized the significance of positive distractions in hospital settings as a key component in improving the environment of cancer centers and supporting the recovery process for young patients.

### 3.3. Hospital Building Description

An empirical investigation was conducted at the King Hussein Cancer Center (KHCC) in Amman, Jordan. Founded in 2001, KHCC is a 13-story hospital located in Jordan’s capital, spanning 108,700 square meters. The facility includes 352 beds, 13 operating rooms equipped with state-of-the-art surgical equipment, and 36 intensive care units. Additionally, KHCC houses a comprehensive laboratory for various tests and a radiation, nuclear medicine, and radiotherapy center with advanced technologies for precise and rapid cancer diagnosis and treatment.

### 3.4. Data Collection Procedures

**Figure 7:**
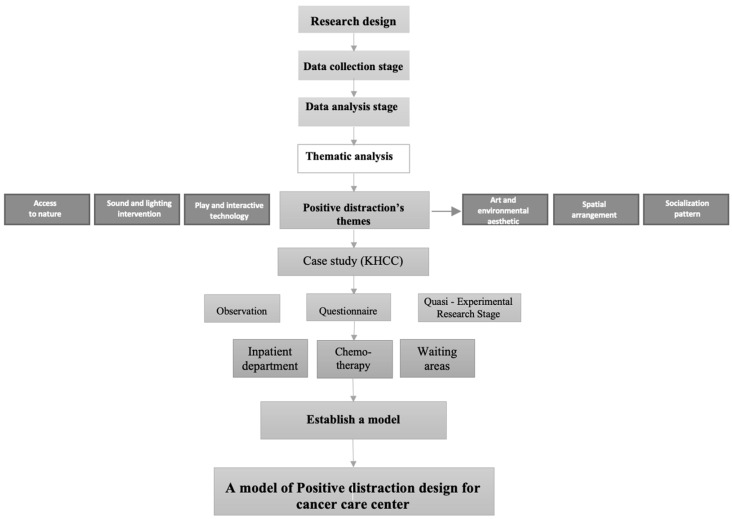
Research structure.

A range of strategies was employed to gather information for measuring and evaluating various aspects of patient experiences at KHCC, as illustrated in Figure 7. The study aimed to assess the overall performance of pediatric patients in a cancer center enriched with positive distractions.

The data collection methods for this study included personal on-site observations, a structured questionnaire distributed to doctors and parents of patients, and a quasi-experimental study involving pediatric patients.

#### 3.4.1. Personal On-Site Observations

Personal observations of the hospital premises were conducted across multiple visits. This assessment was guided by a framework derived from literature analysis and supported by photographic documentation.

#### 3.4.2. Questionnaires with Doctors

Questionnaires with doctors were administered and statistically analyzed. These included questions surrounding satisfaction within an environment equipped with positive distractions. The literature analysis was used to formulate the questionnaire, which was distributed to elucidate children’s patient satisfaction and their visual comfort within the hospital environment.

#### 3.4.3. Quasi-Experimental Design

In this study, pediatric patients were observed to gather their perceptions of six themes of positive distractions in the hospital environment. A quasi-experimental design was applied to compare the impact of these positive distractions on reducing stress and enhancing the overall spatial experience among the children. The aim was to evaluate whether the introduction of positive environmental distractions improved pediatric patients’ behavioral stress responses and mood states at King Hussein Cancer Center (KHCC) in Amman, Jordan. The study compared two different settings: a low-traditional environment without intervention and a high-intervention setting featuring elements such as colorful lighting, art, nature, social interaction and play, and interactive technology.

To assess children’s behavior, various measurements were utilized, starting with baseline data collection on anxiety levels, stress, and overall well-being. In this study, the KIDSCREEN-27 questionnaire was used as the baseline assessment tool, measuring the well-being and stress levels of the children both before and after the introduction of positive distractions. As suggested by Rappaport, research into environmental responses should evaluate how individuals perceive their environment, how they feel about it, what aspects they like or dislike, and their overall attitude toward the environment. For this research, the KIDSCREEN-27 was selected due to its validation and appropriateness for children aged 8 to 18. It measures health-related quality of life (HRQoL) across five dimensions: physical well-being, psychological well-being, autonomy and parent relations, social support and peers, and school environment.

The small sample size of 20 participants was divided into two groups:

- Ten participants in the intervention group, who were exposed to positive distractions in the high-intervention environment.
- Ten participants in the control group, who were in the low-traditional environment without the intervention.

Each participant completed the KIDSCREEN-27 questionnaire before and after the intervention period.

**Figure 8:**
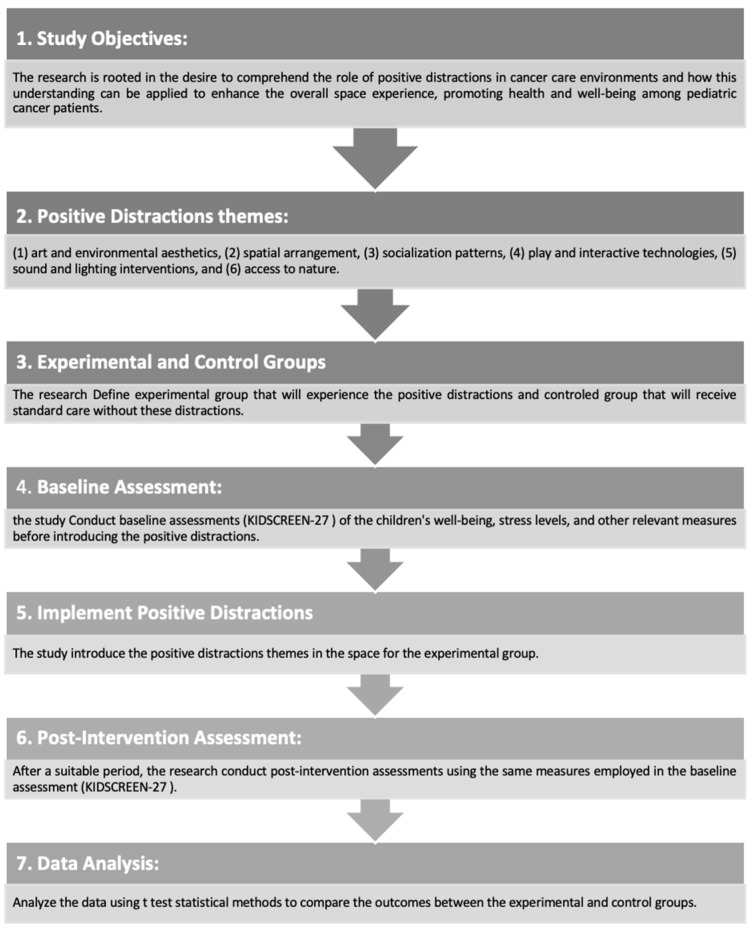
Quasi-experimental research method used in the present study.

Figure 8 illustrates the schematic framework of the quasi-experimental research method used in this study.

### 3.5. Data Analysis

#### 3.5.1. Analysis of On-Site Observation

**Figure 9:**
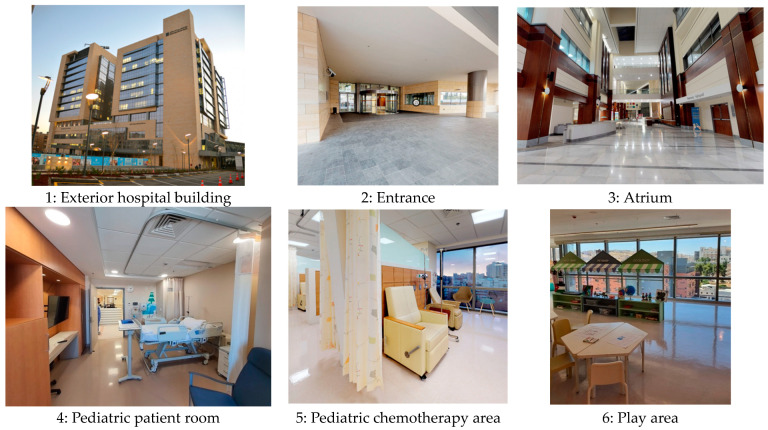
King Hussein Cancer Center in Amman, Jordan, extension part from left to right.

The inpatient room, the chemotherapy area, the waiting rooms, and the play areas were the four places chosen for evaluation at the King Hussein Cancer Center. The researcher concentrated on the crucial aspect of pleasant distractions in each of these areas (Figure 9). To achieve this, the researcher evaluated both the types and amounts of distractions that occurred in each area, taking record of how they affected the experiences and overall health of the patients.

#### 3.5.2. Analysis of the Questionnaire and the Quasi-Experimental Study

Data were entered and statistically analyzed using the Statistical Package for the Social Sciences (IBM SPSS 28) software. An analysis of variance (ANOVA) was conducted to assess differences in perceptions across various demographic and work-related variables, including gender, age, working hours, length of service in the hospital, and department or unit. Following this, the patients’ behaviors in relation to the positive distraction perception results from the quasi-experimental study were measured using both the baseline and post-intervention assessments with the KIDSCREEN-27 questionnaire.

#### 3.5.3. Statistical Analysis

The data collected were summarized and analyzed using various t-tests to compare the results between the intervention and control groups. These groups included a low-traditional environment without the intervention and a high-intervention setting incorporating positive distraction elements.

1. Independent *t*-test: This test was used to compare the post-intervention scores between the intervention and control groups, determining whether there were statistically significant differences in emotional well-being between the two groups.
2. Paired *t*-tests: These tests were employed to compare pre- and post-intervention scores within each group, allowing for an assessment of the impact of the positive distraction intervention on the emotional well-being of the participants.
3. Effect Sizes: To complement the t-test results, effect sizes were calculated to quantify the magnitude of the differences observed. This aided in understanding the practical significance of the findings, beyond mere statistical significance.

## 4. Results Analysis

### 4.1. Respondents’ Characteristics

The target sample for the survey study comprised health care providers and doctors at King Hussein Cancer Center and the families of patients. With regard to the survey of healthcare providers, only qualified hospital personnel were approached for questionnaire answers. Taherdoost’s formula was used to determine the appropriate sample size of health care providers. At the time of this study, in the pediatric oncology department in King Hussein Cancer Center, there were 55 doctors. Substituting these values into the formula, we get:

*N* = [1.96^2^ × 0.5(1 − 0.5)]/[0.05^2^ + (1.96 ^2^ × 0.5(1 − 0.5)/55)] *N* = 48.33

According to Taherdoost’s formula, the target sample size for a total population of 55 with a margin of error of 0.05 is approximately 48.33. Therefore, the target sample size would be 49. The researcher distributed a questionnaire to approximately 49 doctors in the pediatric department.

From the survey conducted among doctors and healthcare providers, 40 respondents participated, of which 30% were female and 70% were male. The respondents were aged between 36 and 55 years and had been working in the healthcare field for a period of 10–18 years. The survey also revealed that most respondents worked between 8 and 15 h per day.

In contrast, only 20 participants from the families of patients completed the survey.

Regarding the quasi-experimental study, the target sample consisted of pediatric inpatients at King Hussein Cancer Center (KHCC). The study involved 20 pediatric patients aged between 8 and 18 years. It was conducted in two primary areas within the hospital: the patient rooms and play areas. Figure 9 illustrates key points, including the main entrance, exterior, and interior details of the building.

### 4.2. Children’s Pediatric Positive Distractions Perception and Impacts

#### 4.2.1. First Phase of Analysis

The aim of this observational study was to examine the different areas of the King Hussein Cancer Center (KHCC) and determine what features allowed patients to experience positive distractions. In order to better understand how these features affected the pediatric patients, the study focused on themes associated with positive distractions, such as nature, art, social patterns, spatial layout, interactive technologies, and lighting and sound interventions.

**Figure 10:**
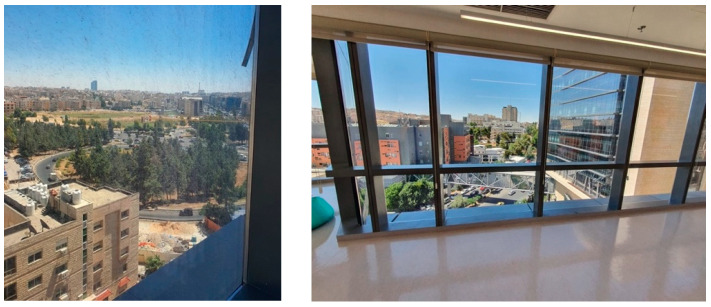
Large windows overlooking greenery in King Hussein Cancer Center in Amman, Jordan.

**Figure 11:**
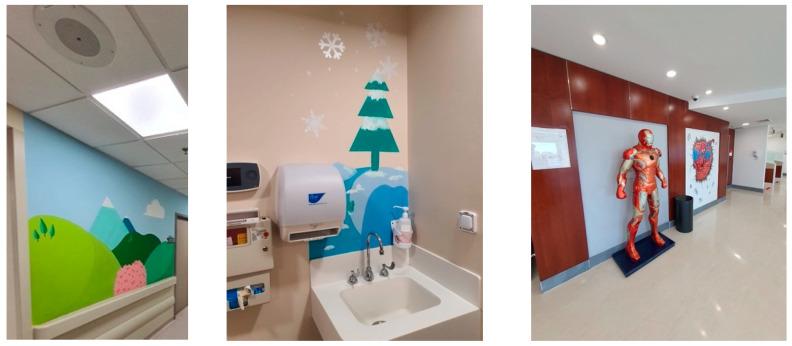
Artworks in King Hussein Cancer Center in Amman, Jordan.

**Figure 12:**
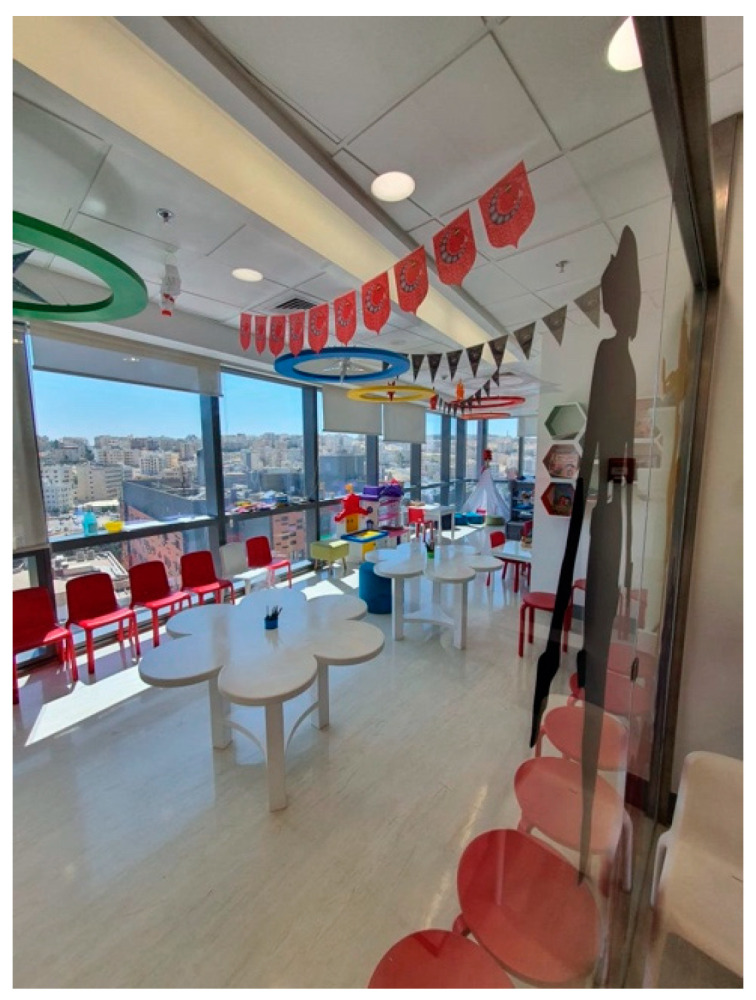
Play area in the King Hussein Cancer Center.

**Figure 13:**
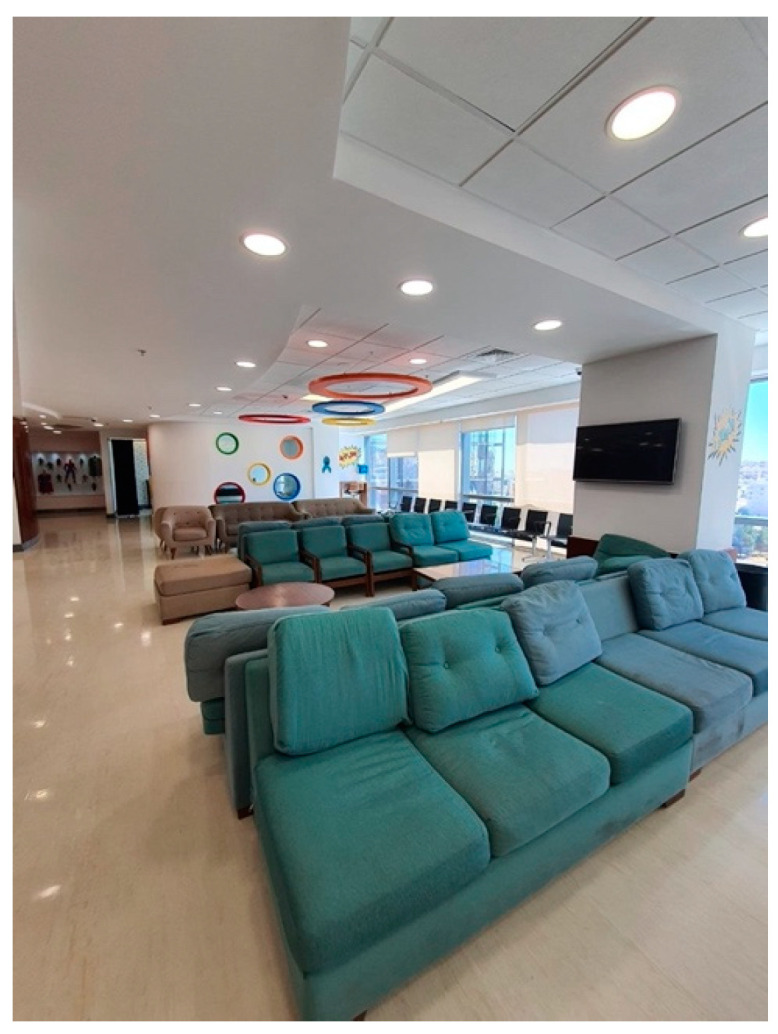
Waiting area in King Hussein Cancer Center in Amman, Jordan.

**Figure 14:**
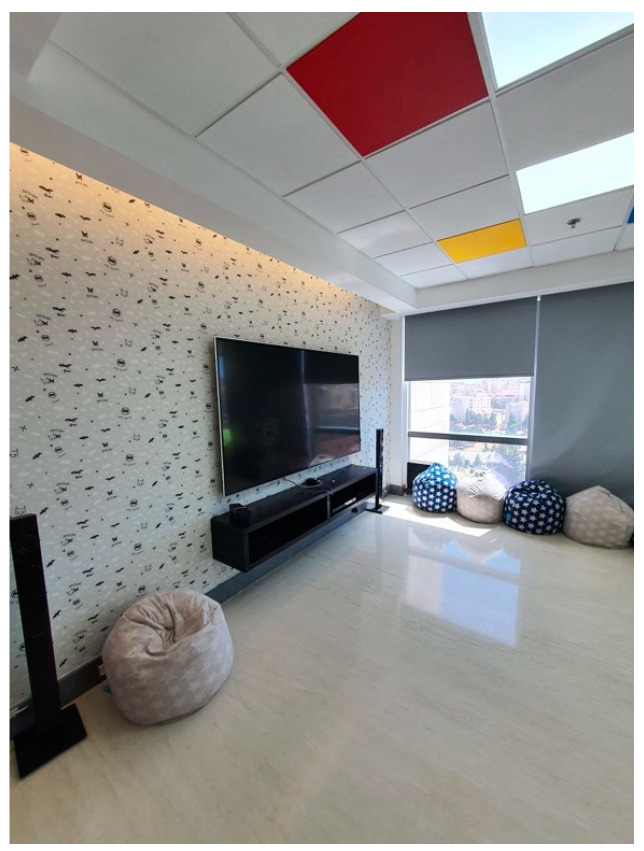
Interactive technologies provided in the waiting area at King Hussein Cancer Center in Amman, Jordan.

**Figure 15:**
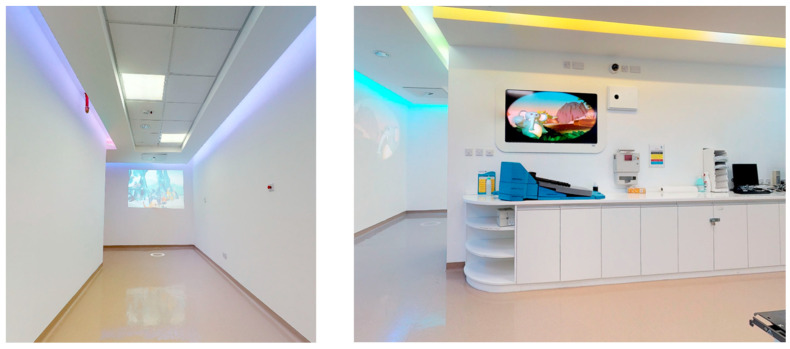
Liner accelerators in King Hussein Cancer Center in Amman, Jordan.

The findings of the observations were as follows:

1. Nature
  - The cancer center had integrated natural features like potted plants and windows with views of greenery, as shown in Figure 10.
  - Patients and visitors were often seen to be drawn towards these spaces, frequently taking moments to gaze out of the windows.
  - The inclusion of natural elements fostered a tranquil ambience, providing a break from the sterile hospital setting and the challenging treatments experienced by cancer patients.
2. Art and the aesthetics of the environment
  - A wide array of artistic expressions could be observed across the facility, encompassing paintings, sculptures, and murals, as shown in Figure 11.
  - The art pieces encompassed a range of subjects, such as landscapes, abstract designs, and inspiring visuals that captured the interest of young patients.
  - Individuals visiting the center, whether patients or guests, interacted with the art installations, taking moments to admire the visual appeal and delve into the interpretive messages conveyed.
3. Patterns of Social Interaction
  - Public spaces, like waiting areas and common rooms, were intentionally created to promote social engagement between patients, families, and medical personnel, as shown in Figure 12 and Figure 13.
  - Seating arrangements facilitated face-to-face communication.
  - Observations indicated that social interactions provided emotional support and a sense of community, reducing feelings of isolation.
4. Spatial arrangement
  - The design of the King Hussein Cancer Center aims to maximize efficiency and ease of access, incorporating prominent signage and navigation tools.
  - Private consultation areas and chemotherapy rooms, as shown in Figure 14, provided peaceful environments for private conversations between patients and medical professionals.
  - The expansive, airy hallways helped create a feeling of openness, reducing any sense of being confined.
5. Interactive technologies:
  - Interactive screens were strategically positioned in waiting areas.
  - Touchscreens and PlayStations were available in waiting areas and playrooms, as shown in Figure 14.
  - Patients interacted with these technologies, exhibiting positive emotions.
6. Audio and Visual Enhancements
  - The lighting scheme integrated natural light whenever feasible, complemented by adaptable artificial lighting as shown in Figure 15, to establish a specific atmosphere.
  - Soft background music enhanced the auditory environment, fostering a calming ambience, despite the presence of musical instruments in designated play zones.
  - Studies have indicated that the strategic use of lighting and sound aids in promoting relaxation and comfort, ultimately leading to a decrease in stress levels.

The study’s observations validated that the areas within the King Hussein Cancer Center were filled with beneficial positive distractions, including nature, art, social designs, spatial organization, interactive technologies, and interventions in lighting and sound. These characteristics helped create a therapeutic setting that catered to the overall needs of patients, promoting a feeling of wellness and resilience amidst the challenges of cancer treatment.

#### 4.2.2. The Second Phase of Analysis

The outcomes of a survey, containing a mix of closed and open-ended queries, carried out with healthcare professionals and family members of patients at King Hussein Cancer Center were analyzed. The survey sought to collect perspectives on the possible impact of positive distractions, including nature, art, social engagements, spatial layout, interactive technologies, and lighting and sound interventions, on pediatric patients receiving cancer therapy. The objective was to explore the perceived effects of positive distractions on children in cancer treatment facilities, as recounted by healthcare professionals and parents of patients at King Hussein Cancer Center.

The first survey was completed by 40 healthcare professionals working in pediatric cancer centers. The demographic breakdown was as follows:

Age: The majority of respondents were between 36 and 55 years old.

Gender: 70% were male, 30% female.

Work Hours: The average number of hours worked per day was 11 h.

Experience: The respondents had an average of 14 years of experience in their current roles.

The findings suggested that the King Hussein Cancer Center was adequately equipped with beneficial distractions and comforting surroundings that aided the overall well-being and stress relief of pediatric patients. Natural light, proximity to nature, and thoughtful design features were key factors in improving the patients’ experience. Nevertheless, there was potential for enhancement by providing more direct access to gardens and reducing environmental stressors even further.

The second survey gathered responses from 20 parents or guardians whose children were currently receiving care at pediatric cancer centers. Here was the demographic breakdown:

Relationship to Child:

Mother: 65%

Father: 30%

Grandmother: 2%

Grandfather: 1%

Other relatives or legal guardians: 2%

Age:

18–24: 3%

25–34: 20%

35–44: 45%

45–54: 20%

55–64: 10%

65–74: 2%

The findings indicated that the King Hussein Cancer Center was creating environments that offered positive distractions, contributing to the comfort and stress reduction of child patients. Elements like natural light, engaging artwork, and spaces for social interaction were highlighted as beneficial. However, there was room for improvement, especially in providing consistent access to outdoor areas and reducing noise disruptions. In conclusion, a well-designed environment could greatly improve the hospital experience for children receiving cancer treatment.

#### 4.2.3. The Third Phase of Analysis

**Figure 16:**
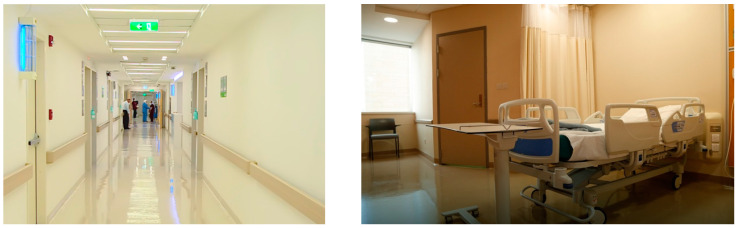
A low-intervention traditional environment in the original building of KHCC.

**Figure 17:**
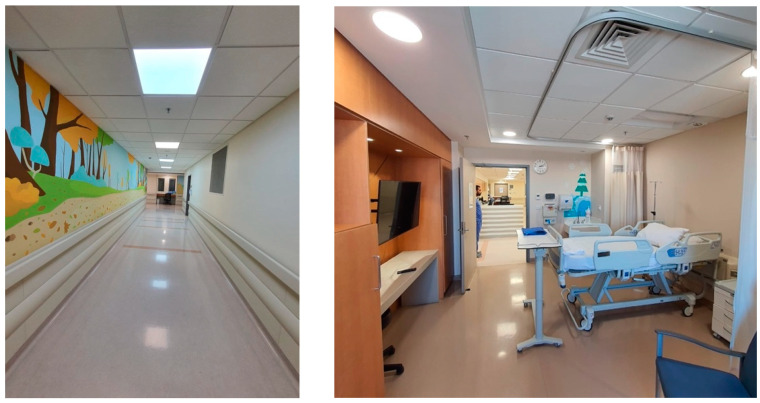
A high-intervention operating mode with positive distraction elements in the newly constructed expansion building of KHCC in Amman, Jordan.

The study compared two groups situated in distinct environments: a control group in a low-intervention setting and an intervention group in a high-intervention setting with positive distraction elements. The low-intervention environment was located in the original building of King the Hussein Cancer Center, while the high-intervention environment was situated in the newly constructed expansion building. The high-intervention environment incorporated various positive distraction elements, including colorful lighting, sound interventions, art, nature, social interaction, spatial arrangement, and interactive technology. Figure 16 and Figure 17 provide visual details of these environments.

A quasi-experimental study was conducted to evaluate the effects of positive distractions on behavioral stress responses and mood states in pediatric patients. The KIDSCREEN-27 questionnaire was used to assess levels of well-being and stress in pediatric patients aged 8 to 18, both before and after the implementation of positive distractions.

##### Descriptive Statistics

Table 2 presents the mean and standard deviation (SD) for each dimension of the KIDSCREEN-27 questionnaire, both before and after the intervention, for both the control and intervention groups.

**Figure 18:**
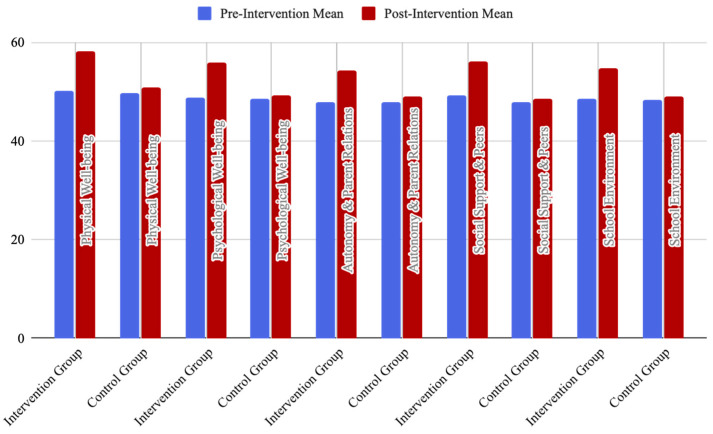
The mean scores for both the intervention and control groups, comparing pre-intervention and post-intervention data.

The following chart (Figure 18) illustrates the mean scores for both the intervention and control groups, comparing pre-intervention and post-intervention data.

##### Statistical Analysis

1. Paired *t*-Test: Pre-Post Comparisons within Groups
  - Intervention Group: Significant improvements were observed across all five dimensions of the KIDSCREEN-27, with *p*-values less than 0.05:
    o Physical well-being: t (9) = −4.27, *p* = 0.002
    o Psychological well-being: t (9) = −5.11, *p* = 0.001
    o Autonomy and parent relations: t(9) = −4.59, *p* = 0.001
    o Social support and peers: t (9) = −4.89, *p* = 0.001
    o School environment: t (9) = −4.37, *p* = 0.002
  - Control Group: No significant changes were found in any of the dimensions, with *p*-values greater than 0.05:
    o Physical well-being: t (9) = −1.28, *p* = 0.23
    o Psychological well-being: t (9) = −0.91, *p* = 0.38
    o Autonomy and parent relations: t (9) = −1.12, *p* = 0.29
    o Social support and peers: t (9) = −1.01, *p* = 0.33
    o School environment: t (9) = −1.15, *p* = 0.28
2. Independent t-Test: Between-Group Comparisons Post-Intervention
  Significant differences were found between the intervention and control groups across all dimensions, with *p*-values less than 0.05:
    o Physical well-being: t (18) = 3.89, *p* = 0.001
    o Psychological well-being: t (18) = 3.79, *p* = 0.002
    o Autonomy and parent relations: t (18) = 3.54, *p* = 0.002
    o Social support and peers: t (18) = 3.71, *p* = 0.002
    o School environment: t (18) = 3.59, *p* = 0.002
3. Effect Size (Cohen’s d):

Large effect sizes were observed in all dimensions for the intervention group, indicating a substantial impact of positive distractions on pediatric patients’ well-being (Table 3).

##### Interpretation of Results

- Positive Outcomes: The intervention group demonstrated significant improvements across all five dimensions of the KIDSCREEN-27 compared to the control group. These results suggested that the positive distractions implemented in the high-intervention environment had a beneficial impact on the health-related quality of life (HRQoL) of pediatric cancer patients.
- Non-significant Outcomes: The control group, which was in a low-traditional environment without positive distractions, did not exhibit significant changes in any of the dimensions. This lack of significant change reinforced the likelihood that the observed improvements in the intervention group were attributable to the positive distractions.
- Patients in the intervention group reported significantly higher satisfaction levels compared to the control group. Key measures showing significant improvement included physical well-being and overall health perception (see Table 2 and Figure 18).

## 5. Discussion

This study aimed to identify specific design components that served as beneficial distractions and evaluated their effects on the emotional and psychological well-being of patients. The examination of King Hussein Cancer Center (KHCC) highlighted several significant themes of positive distractions:

- Natural Elements: Patients frequently reported that gardens and views of nature provided comfort and relaxation. This finding aligns with Ulrich’s theory of supportive design, which posits that natural elements can reduce stress and support recovery. This observation is consistent with prior research demonstrating the healing effects of nature exposure in healthcare environments.
- Artistic Features: Artwork and visually appealing surroundings were noted to enhance tranquility and mood. This supports the idea that visual and aesthetic components can serve as positive distractions by shifting focus away from stress and discomfort. The variety of art styles also catered to different patient preferences, enriching the overall patient experience.
- Interactive Features: Interactive installations, such as light displays, engaged patients and offered cognitive stimulation. These features effectively redirected attention from treatment-related anxiety and discomfort, corroborating insights from environmental psychology that suggest that engagement and diversion can alleviate perceived pain and distress.
- Community Areas: Spaces designed for social interaction, such as communal lounges and activity rooms, facilitated social support and reduced feelings of loneliness. These areas promoted a sense of community and provided emotional comfort, further enhancing the overall well-being of patients.

These findings have contributed to the growing body of evidence supporting the integration of therapeutic design elements in healthcare environments. They underscored the importance of creating spaces that offer comfort, engagement, and social support to enhance patient experiences. The comprehensive theoretical framework developed through this research serves as a valuable tool for designing pediatric cancer care settings that promoted healing. By incorporating psychological, physical, and societal perspectives, the framework offers a holistic approach to improving the overall care experience. It provides guidance for architects, healthcare designers, and policymakers in creating environments that not only address medical needs, but also foster a comforting and supportive atmosphere for children undergoing treatment.

The findings underscore the importance of incorporating positive distractions into healthcare facility design, particularly in cancer treatment centers. By integrating natural elements, art, interactive features, and community spaces, healthcare environments can create more supportive and healing atmospheres for patients. This approach contributes to a growing body of evidence suggesting that therapeutic design elements play a crucial role in improving patient outcomes and experiences. Table 4 outlines key design aspects for pediatric cancer care centers and their positive impact on patients.

However, this study has limitations, including its focus on a single cancer center, which may restrict the generalizability of the results. Additionally, the reliance on subjective assessments of emotional and psychological effects based on patient feedback may introduce bias. Future research would benefit from a larger sample size and the inclusion of objective metrics, such as physiological stress indicators, to provide a more comprehensive evaluation of the impacts of positive distractions. Future studies should also explore the long-term effects of positive distractions on patient outcomes, including stress levels, treatment adherence, and overall satisfaction. Investigating which specific characteristics of nature, art, and interactive components are most effective as positive distractions could further enhance our understanding and application of these design elements in pediatric cancer care centers.

## 6. Conclusions

This research sought to deepen our understanding of the role of positive distractions in cancer care settings, particularly focusing on improving the overall experience and well-being of pediatric patients. The study effectively established a theoretical framework highlighting the importance of positive distractions in enhancing the care environment for young patients. By integrating principles from supportive design, evidence-based design for healthcare, and attention restoration theory, the research provided a robust foundation for identifying and implementing design features that contributed to the well-being of children undergoing cancer treatment.

The identified themes of positive distractions—including nature and green spaces, art and aesthetics, interactive elements, and social spaces—demonstrated substantial potential in improving the well-being of pediatric cancer patients. These findings have contributed to the growing body of evidence supporting the integration of therapeutic design elements in healthcare environments. They underscore the importance of creating spaces that offer comfort, engagement, and social support to enhance patient experiences.

The comprehensive theoretical framework developed through this research serves as a valuable tool for designing pediatric cancer care settings that promoted healing. By incorporating psychological, physical, and societal perspectives, the framework offers a holistic approach to improving the overall care experience. It provides guidance for architects, healthcare designers, and policymakers in creating environments that not only address medical needs, but also foster a comforting and supportive atmosphere for children undergoing treatment.

While the study highlighted the potential benefits of positive distractions in improving emotional well-being, it also emphasized the need for empirical data linking these factors to clinical outcomes, such as healing times. Although the current study offered valuable insights, future research should integrate quantitative measures to more rigorously assess the impact of positive distractions on clinical outcomes. Prospective studies could track healing times and recovery rates among patients exposed to these interventions, and analyzing physiological indicators could provide a deeper understanding of the underlying mechanisms influencing health outcomes.

In summary, the findings suggested that positive distractions play a crucial role in pediatric care by enhancing emotional well-being. However, further research is necessary to determine the generalizability of these results to other settings and to establish stronger empirical evidence supporting their implementation in pediatric care environments.

## Figures and Tables

**Table 1:** The connection between the design factors of positive distractions in healthcare and the resulting health outcome.

Positive Distraction’s Themes	Design Elements	Health Outcomes
Theme 1: Art and the aesthetics of the environment.	Nature art Ocean and beach views Preferred pale to mid color ranges and mid blue- green colors Glitter or shine, metals and stars, and striped materials	Associated with the pediatric quality of life and parent and staff satisfaction.
Theme 2: Spatial Arrangement	Public central atrium spaces	Enhanced opportunities to socialize and was associated with young patients’ emotional status. It was a place to see, be seen, and be involved in the social life of the hospital.
Theme 3: patterns of social interaction	Technology Positive social Spatial distractions Entertainment activities	Support children’s psychosocial well-being when in confined healthcare spaces.
Theme 4: Interactive technologies	Digital displays (e.g., television [tv] screens) non-touchable medium virtual reality ambient environment Multisensory stimuli display Computer-mediated augmented environments Nature-themed content (e.g., nature, aquarium, animals, and zoo)	Included reduced pain and distress during medical procedures, reduced anxiety, reduced arousal, and calm behaviors and tranquility among pediatric patients.
Theme 5: Audio and visual enhancements	Natural sound intervention. Music. Using colorful ambient lighting to match the theme of animation and art.	Therapeutic by reducing noise, enhancing tranquility, and calming adolescent patients’ behaviors in waiting areas.
Theme 6: Nature	A variety of high-quality seats and child-scaled furniture Improved accessibility and circulation More trees, greenery, and shade A pleasant microclimate Playful features that encourage play activities Security and privacy	Place of restoration and healing, increased consumer satisfaction, enhanced emotional respite for visitors, and reduced pain and distress.

**Table 2:** KIDSCREEN-27 Dimensions and the Mean Scores.

Dimension	Group	Pre-Intervention Mean (SD)	Post Intervention Mean (SD)
Physical well-being	Intervention group	50.2 (4.5)	58.3 (5.1)
	Control group	49.7 (4.8)	50.9 (5.0)
Psychological well-being	Intervention group	48.7 (5.0)	55.9 (4.8)
	Control group	48.5 (4.6)	49.2 (4.7)
Autonomy and parents relations	Intervention group	47.8 (5.3)	54.2 (5.4)
	Control group	48.0 (5.2)	49.1 (5.3)
Social support and peers	Intervention group	49.3 (4.7)	56.1 (5.0)
	Control group	47.9 (4.9)	48.5 (5.1)
School environment	Intervention group	48.5 (4.9)	54.8 (5.2)
	Control group	48.3 (5.0)	49.0 (5.2)

**Table 3:** The KIDSCREEN-27 dimensions with (Cohen’s d) effect size.

Dimension	Cohen’s d
Physical Well-being	1.35
Psychological Well-being	1.21
Autonomy & Parent Relations	1.14
Social Support & Peers	1.19
School Environment	1.17

**Table 4:** Framework table outlining key design aspects for pediatric cancer care centers and their positive impact on patients.

Design Aspects	Description	Positive Impacts on Patient
Nature-Inspired Design	Incorporation of natural elements such as greenery, natural light, and views of nature	Reduce stress and anxiety, improved mood and cognitive function
Play and recreational space	Dedicated areas with age-appropriate toys, games, and activities for play and social interaction.	Enhanced emotional expression, socialization, and coping mechanism
Art and music therapy spaces	Studios equipped with art supplies, musical instruments, and interactive installations.	Alleviation of pain, anxiety, and depression, enhanced emotional expression and communication.
Technology integration	Integration of interactive screens, VR experiences, gaming consoles, and telecommunication facilities.	Distraction from medical procedures, education, entertainment, and communication.
Social and community spaces	Communal areas for group activities, family gatherings, and support group meetings.	Enhanced sense of belonging, peer support, and emotional connection.
Sensory stimulation environment	Multisensory elements like aromatherapy, tactile surfaces, soothing sounds, and interactive lighting	Engagement of multiple senses for distraction from pain and discomfort.
Flexibility and personalization	Adaptable spaces with flexible furniture arrangements, movable partitions, and adjustable lighting.	Personalized environments tailored to individual preferences and changing needs.
Safety and comfort	Use of durable, easy-to-clean materials that meet healthcare standards and regulations.	Ensured safety and comfort for patients, families, and healthcare staff.

## Data Availability

Data supporting the reported results can be requested from the corresponding author.
